# Bias in machine learning models can be significantly mitigated by careful training: Evidence from neuroimaging studies

**DOI:** 10.1073/pnas.2211613120

**Published:** 2023-01-30

**Authors:** Rongguang Wang, Pratik Chaudhari, Christos Davatzikos

**Affiliations:** ^a^Department of Electrical and Systems Engineering, University of Pennsylvania, Philadelphia, PA 19104; ^b^Center for Biomedical Image Computing and Analytics, University of Pennsylvania, Philadelphia, PA 19104; ^c^Department of Computer and Information Science, University of Pennsylvania, Philadelphia, PA 19104; ^d^Department of Radiology, Perelman School of Medicine, University of Pennsylvania, Philadelphia, PA 19104

**Keywords:** machine learning, algorithmic bias, neuroscience, MRI, heterogeneity

## Abstract

Despite the great promise that machine learning has offered in many fields of medicine, it has also raised concerns about potential biases and poor generalization across genders, age distributions, races and ethnicities, hospitals, and data acquisition equipment and protocols. In the current study, and in the context of three brain diseases, we provide evidence which suggests that when properly trained, machine learning models can generalize well across diverse conditions and do not necessarily suffer from bias. Specifically, by using multistudy magnetic resonance imaging consortia for diagnosing Alzheimer’s disease, schizophrenia, and autism spectrum disorder, we find that well-trained models have a high area-under-the-curve (AUC) on subjects across different subgroups pertaining to attributes such as gender, age, racial groups and different clinical studies and are unbiased under multiple fairness metrics such as demographic parity difference, equalized odds difference, equal opportunity difference, etc. We find that models that incorporate multisource data from demographic, clinical, genetic factors, and cognitive scores are also unbiased. These models have a better predictive AUC across subgroups than those trained only with imaging features, but there are also situations when these additional features do not help.

Machine learning models have shown great promise for precision diagnosis, treatment prediction, and a number of other clinical applications (1). There is increasing interest in building systems where such models can aid human experts in clinical settings. However, there are some key challenges to achieving this goal (2). Clinical data are highly heterogeneous. For neurological disorders such as Alzheimer’s disease, it stems not only from diverse anatomies, overlapping clinical phenotypes, or genomic traits of different subjects, but also from operational, demographic, and social aspects such as data acquisition protocols (3) and paucity of data for minorities. As a consequence, machine learning models often have poor reproducibility across different subgroups in the population.

We focus on one particular aspect of this issue, namely that models make biased predictions, i.e., they have different AUCs for different genders, age and racial groups, and cohorts from different clinical studies. This has received wide attention (4–10). For example, a model trained on X-ray images consistently made inaccurate predictions on underrepresented genders when the training data were imbalanced (4). Similarly, for the classification of chest X-ray pathologies, minority groups such as female African Americans and patients with low socioeconomic status are prone to be incorrectly diagnosed as healthy (6). Such results have raised concerns about whether machine learning models can provide unbiased predictions and whether they can be deployed in clinical settings eventually.

We provide results from relatively large and diverse datasets pertaining to three neurological disorders, namely Alzheimer’s disease (AD), schizophrenia (SZ), and autism spectrum disorder (ASD), which partly alleviate these concerns. We build models using magnetic resonance (MR) imaging features along with demographic, clinical, genetic factors, and cognitive scores, from large-scale consortia—iSTAGING (11) for AD, PHENOM (12) for SZ, and ABIDE (14) for ASD; (*SI Appendix*). There are large imbalances in this dataset, e.g., 13% and 37.4% female subjects, respectively, in ABIDE and PHENOM and 70.6% European Americans, 8.8% African Americans, and 1.5% Asian Americans in iSTAGING. A large body of literature has studied confounders such as gender, age, race, and image acquisition protocol for these data (15, 16). We show that, when trained with appropriate data-preprocessing techniques and hyperparameter tuning, machine learning models do not have biased predictions across different subgroups. In contrast, a baseline deep neural network provides accurate predictions on average across the population but suffers from bias.

## Results

### We Demonstrate Bias in Baseline Models in Spite of Their High Accuracy.

**Fig. 1.**
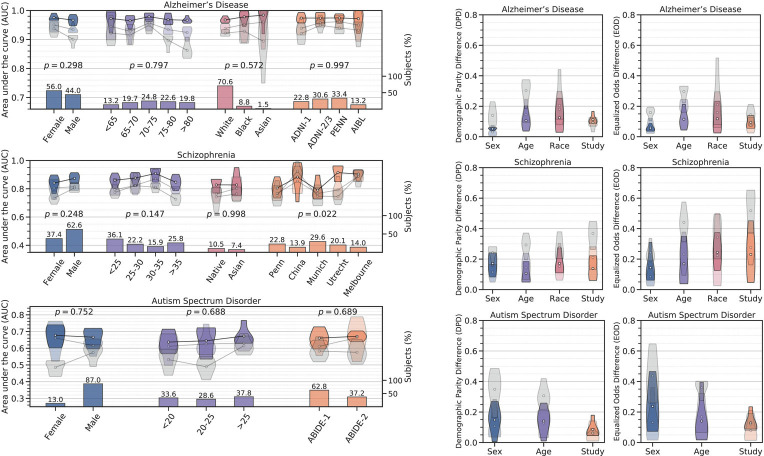
(*Left*) Evaluating machine learning models on subjects from different gender, age, racial groups and clinical studies. For Alzheimer’s disease, schizophrenia, and autism spectrum disorder, using data from different studies (e.g., ADNI-1, ADNI-2/3, PENN, and AIBL for Alzheimer’s disease), we built an ensemble that uses data from multiple sources (MR imaging features, demographic, clinical variables, genetic factors, and cognitive scores); (*SI Appendix*). This ensemble was trained using preprocessing and hyperparameter optimization discussed in *Materials and Methods*. We also trained an ensemble using only imaging features. Bar plots denote the size of the subgroup/study (%); in many cases, there is a strong imbalance in the data. Violin plots denote the test AUC on five different held-out subsets of data. Solid colors indicate that models used all features, while translucent colors indicate that models were trained only on imaging features. Translucent gray denotes the AUC of a baseline deep network (without appropriate preprocessing and hyperparameter tuning). White dots denote the average AUC of each subgroup/study. For the ensemble trained on multisource data, the *P*-values shown in the figure indicate that we cannot reject the null hypothesis that the AUC for different subgroups has the same mean (at significance level < 0.01). This is not the case for the baseline deep network. (*Right*) Fairness assessment with respect to sensitive attributes. Violin plots denote the test demographic parity differences (DPD) and equalized odds difference (EOD) (20) on five different held-out subsets of data. White dots denote the average metric of each subgroup/study. A model is perfectly fair if the fairness disparity (DPD or EOD) is zero. Ensemble models (imaging features or multiple sources) have lower disparities for all sensitive attributes in both metrics (differences in the two are not statistically significant) as compared to the baseline deep network (all *P*-values < 0.01). It has been noticed that different fairness metrics are often incompatible. We therefore also assessed fairness using three other metrics: equal opportunity difference (EO), predictive parity difference (PPD), and generalized entropy index (GEI). In all cases, ensemble models show lower disparities compared to baseline models, except the GEI metric for autism spectrum disorder (*SI Appendix*).

The AUC of a deep network on held-out data (imaging features) is 0.924 ± 0.008 for AD, 0.806 ± 0.036 for SZ, and 0.572 ± 0.031 for ASD. These numbers are comparable to published results (17–19). As Fig. 1 shows, for all three disorders, we find large discrepancies in the AUC on different subgroups for all attributes. For example, for AD, the model has a higher AUC on females than males (*P* and Cohen’s *f*-value 2.10×10^−3^, 1.13); for SZ, the model predicts more accurately for males than females (*P* and *f*-value 7.47×10^−3^, 1.26). One may be inclined to hypothesize that this bias in the AUC comes from one subgroup having a larger sample size than the other. This hypothesis does not hold when predictions are stratified by age. AD subjects older than 80 y, SZ subjects older than 35 y, and ASD subjects younger than 20 y have a lower AUC (*P*-values are 1.93×10^−3^, 6.31×10^−3^, and 1.07×10^−3^, and *f*-values are 1.12, 1.06, and 1.02, respectively) even if these subgroups are not the ones with the smallest sample size. The baseline deep network is biased (*P*-value < 0.01) for all three disorders except in four cases: race and clinical studies in AD, race in SZ, and clinical studies in ASD.

### We Demonstrate How This Bias Is Significantly Reduced When the Models Are Carefully Trained.

The AUC of the ensemble (*SI Appendix*) on held-out data (imaging features) is 0.935 ± 0.014 for AD, 0.811 ± 0.026 for SZ, and 0.631 ± 0.028 for ASD; all three are slightly better than that of the baseline deep network. As Fig. 1 shows visually, the AUC of the ensemble is more consistent. For all three neurological disorders, for all attributes, we cannot reject the hypothesis that AUCs for different subgroups have the same mean (at significance level < 0.01). In other words, the ensemble does not exhibit a bias, up to statistically indistinguishable levels. It is remarkable that this holds even with an extreme imbalance in the data, e.g., 87% males and 13% females in ASD.

Using the same preprocessing and hyperparameter tuning methodology as that of the ensemble, the bias of the baseline deep network can be improved. We obtained an AUC of 0.924 ± 0.013 on AD, 0.762 ± 0.023 on SZ, and 0.565 ± 0.063 on ASD. These numbers, except for SZ, are about the same as that of the baseline deep network. The *P*-values (and Cohen’s *f*-values in parentheses) for there being bias across subgroups now are 0.086 (sex, 0.69), 0.379 (age, 0.47), 0.976 (race, 0.06), and 0.578 (study, 0.36) for AD; 0.710 (sex, 0.14), 0.173 (age, 0.59), 0.776 (race, 0.10), and 4.77×10^−4^ (study, 1.27) for SZ; 0.321 (sex 0.37), 0.254 (age, 0.51), and 0.818 (study, 0.08) for ASD. The deep network trained with better data preprocessing achieves a similar average AUC as that of the baseline network, but its predictions are not biased (significance level < 0.01), except across clinical studies in SZ, which could be due to differences in clinical characteristics across patient cohorts.

### We Also Demonstrate How This Bias Is Significantly Reduced When Models Are Trained on Multisource Data.

We next trained the ensemble using demographic features, clinical variables, genetic factors, and cognitive scores in addition to MR imaging features. The AUC of this multisource ensemble on held-out data is 0.968 ± 0.009 for AD, 0.866 ± 0.024 for SZ, and 0.663 ± 0.034 for ASD. All three values are better than the corresponding ones for the ensemble trained only on structural MR imaging features (*P*-value < 3.61×10^−3^ for all three). Therefore, using multisource data improves the average AUC of models for these three disorders. This ensemble also makes unbiased predictions across different subgroups; the *P*-values (and *f*-values in parentheses) are 0.298 (sex, 0.39), 0.797 (age, 0.29), 0.572 (race, 0.31), and 0.997 (study, 0.05) for AD; 0.248 (sex, 0.44), 0.147 (age, 0.62), 0.998 (race, 0.00), and 0.022 (study, 0.86) for SZ; and 0.752 (sex, 0.12), 0.688 (age, 0.25), and 0.689 (study, 0.15) for ASD.

A two-way ANOVA as to whether the improved average AUC translates to an improved AUC for subgroups pertaining to each attribute has *P*-values 9.26×10^−3^ (sex), 1.90×10^−2^ (age), 0.465 (race), and 0.636 (study) for AD; 0.250 (sex), 3.31×10^−2^ (age), 0.713 (race), and 7.86×10^−3^ (study) for SZ; and 4.24×10^−2^ (sex), 0.237 (age), and 0.681 (study) for ASD. At a significance level of 0.01, we find that using multisource data improves the AUC of the ensemble as compared to using features from only imaging features for subgroups pertaining to sex in AD and clinical studies in SZ, but this does not hold for other cases.

## Discussion

As machine learning is being applied to problems in the clinical sciences, there is an increasing amount of discussion on bias in particular and ethical issues in general (7). There is also a large amount of recent work on identifying biases (4, 6, 8) and developing techniques to mitigate them (5). We showed that when models are trained using appropriate data preprocessing and hyperparameter optimization techniques, their predictions need not be biased for neuroimaging data. Baseline models, e.g., deep networks, that do not use this preprocessing and hyperparameter optimization predict accurately on average but can be biased. We have also checked that training with sufficient data preprocessing but without adequate hyperparameter tuning produces unbiased models but with lower AUCs (*SI Appendix*). Our results do not diminish the value of the existing work on bias. Instead, they provide evidence that we might be able to develop unbiased machine learning-based diagnostic models and deploy them in practice in the future. However, in some situations, bias cannot be removed by preprocessing and model selection because the source of the bias may be somewhere else, e.g., different rates of misdiagnosis (21).

The machine learning literature has well-established safeguards against poor generalization. Disregarding these procedures can lead to biased predictions. Our results indicate that a rigorous preprocessing, training, and evaluation methodology can give models that do not suffer from biased predictions in neuroimaging applications. This has also been noticed recently (9), but we have demonstrated this phenomenon more exhaustively and across three different neurological disorders. Our work can therefore provide a benchmark for automated neuroimaging-based diagnostic systems.

Balanced datasets are desirable for building unbiased models (22). But it is extremely difficult to obtain balanced data. There are long-standing problems in recruiting volunteers across gender, age, and race. For example, females, minority ethnicity groups, and older subjects are less likely to participate in clinical trials (23). Even for balanced datasets, trained models may still be biased due to unobserved confounders, e.g., severity of the disease or genetic factors. Recent studies have therefore argued for training models on extensive multisource neuroimaging datasets (24). Our results agree with such motivations—large-scale cohorts of multisource data can enable training robust and unbiased models and also enable thorough evaluation.

## Materials and Methods

We use 3D magnetic resonance images along with demographic (gender, age, race, education level, marital status, employment status, handedness, and smoker), clinical (diabetes, hypertension, hyperlipidemia, systolic/diastolic blood pressure, and body mass index), and genetic factors (apolipoprotein E (APOE) alleles 2, 3, and 4) and cognitive scores from three large consortia—iSTAGING (13) for AD, PHENOM for SZ, and ABIDE for ASD; *SI Appendix*. We use a standard processing pipeline (*SI Appendix*) to compute imaging features from T1-weighted MR images. All accuracies are calculated using five independent held-out test sets.

### Feature Preprocessing Pipeline.

Some features, predominantly clinical and genetic factors and cognitive scores, are sparsely populated; *SI Appendix*. Continuous-valued features are normalized to have zero mean and unit variance after median imputation; quantile normalization is used for features with skewed distributions. For categorical features, we introduce an “unknown” category for missing values, and for each feature with missing values, we introduce an additional Boolean feature which indicates whether the value was missing. No harmonization tools (3) are used.

We use AutoGluon (25) for training all models, for building ensembles using bagging, boosting, and stacking and for performing hyperparameter search. The baseline deep network has three fully connected layers and is trained using data that is normalized to zero mean and unit variance after dropping missing values, without feature preprocessing.

## Supplementary Material

Appendix 01 (PDF)

## Data Availability

Previously published data were used for this work; Jack Jr., R. Clifford, et al., The Alzheimer’s disease neuroimaging initiative (ADNI): MRI methods. J. Magn. Resonance Imaging 27, 685–691 (2008).
