# Supplementary material for: Bias in machine learning models can be significantly mitigated by careful training: Evidence from neuroimaging studies
Source: Proc Natl Acad Sci U S A. 2023 Jan 30;120(6):e2211613120. doi: 10.1073/pnas.2211613120 (PMC9962919; doi:10.1073/pnas.2211613120)
Supplement: Supplementary file 1 — Appendix 01 (PDF) [file pnas.2211613120.sapp.pdf]

Supporting Information for

Bias in Machine Learning Models Can Be Significantly Mitigated by Careful Training: Evidence from Neuroimaging Studies

Rongguang Wang, Pratik Chaudhari and Christos Davatzikos

Corresponding Authors: Pratik Chaudhari, Christos Davatzikos.
E-mail: pratikac@seas.upenn.edu, christos.davatzikos@pennmedicine.upenn.edu

This PDF file includes:

Supporting text
SI References

Supporting Information Text

Data

We use 3D magnetic resonance (T1-weighted) images along with demographic (gender, age, race, education level, marital status, employment status, handedness, smoker), clinical (diabetes, hypertension, hyperlipidemia, systolic/diastolic blood pressure and body mass index), genetic factors (apolipoprotein E (APOE) alleles 2, 3 and 4), and cognitive scores (mini-mental state exam (MMSE), full-scale intelligence quotient (FIQ), verbal intelligence quotient (VIQ), and performance intelligence quotient (PIQ)) from three large consortia—iSTAGING (1) for AD, PHENOM (2–4) for SZ, and ABIDE (5) for ASD. The subset of iSTAGING data used here consists of multiple clinical studies: AD Neuroimaging Initiative (ADNI) (6), Penn Memory Center cohort (PENN), and Australian Imaging, Biomarkers and Lifestyle (AIBL) (7). In PHENOM scans are acquired from five different sites namely Penn (United States), China, Munich, Utrecht, and Melbourne. All tasks in this study are a binary classification problem with two labels (healthy controls and patients). We only use the baseline (first time point) scans from each cohort; all follow-up sessions are excluded. This way there is no data leakage for the same participant between training and test sets. For AD, we select stable cognitive normal (CN) and AD patients based on each participant's longitudinal diagnosis status. We only include subjects who were diagnosed as CN or AD at the baseline and stayed stable during the follow-up sessions.

iSTAGING. The consortium consists of four datasets including ADNI-1 (22.81%), ADNI-2 (30.58%), PENN (33.40%), and AIBL (13.22%). There are in total 781 controls and 815 patients where 173 (controls)/191 (patients) in ADNI-1, 261/227 in ADNI-2/3, 228/305 in PENN, and 119/92 in AIBL. 56.02% of the participants are female and 43.98% of them are male; 10.90% (female)/11.90% (male) in ADNI-1, 15.66%/14.91% in ADNI-2/3, 21.37%/12.03% in PENN, and 8.08%/5.14% in AIBL. The participants are distributed in age ranges 0–65 (13.16%), 65–70 (19.67%), 70–75 (24.81%), 75–80 (22.56%), and over 80 (19.80%) years old; 1.50% (0–65)/1.94% (65–70)/7.27% (70–75)/6.52% (75–80)/5.58% (> 80) in ADNI-1, 3.51%/8.15%/6.95%/6.89%/5.08% in ADNI-2/3, 6.33%/7.02%/6.83%/6.58%/6.64% in PENN, and 1.82%/2.57%/3.76%/2.57%/2.51% in AIBL. 70.61% of the participants are White, 8.77% of them are Black, and 1.50% of them are Asian; 21.18% (White)/1.19% (Black)/0.31% (Asian) in ADNI-1, 16.98%/0.88%/0.56% in ADNI-2/3, 25.13%/6.70%/0.63% in PENN, and 7.33% (White) in AIBL.

PHENOM. The consortium consists of five datasets including Penn (22.28%), China (13.94%), Munich (29.64%), Utrecht (20.12%), and Melbourne (14.03%). There are in total 563 controls and 456 patients where 131 (controls)/96 (patients) in Penn, 76/66 in China, 157/145 in Munich, 115/90 in Utrecht, and 84/59 in Melbourne. 37.39% of the participants are female and 62.61% of them are male; 11.87% (female)/10.40% (male) in Penn, 6.77%/7.16% in China, 7.75%/21.88% in Munich, 6.97%/13.15% in Utrecht, and 4.02%/10.01% in Melbourne. The participants are distributed in age ranges 0–25 (36.11%), 25–30 (22.18%), 30–35 (15.90%), and over 35 (25.81%) years old; 5.79% (0–25)/6.28% (25–30)/3.53% (30–35)/6.67% (> 35) in Penn, 4.91%/2.36%/2.16%/4.51% in China, 9.42%/7.26%/5.99%/6.97% in Munich, 10.11%/4.12%/2.85%/3.04% in Utrecht, and 5.89%/2.16%/1.37%/4.61% in Melbourne. 10.50% of the participants are Native and 7.36% of them are Asian; 10.50% (Native)/7.36% (Asian) in Penn.

ABIDE. The consortium consists of two phases including ABIDE-1 (62.78%) and ABIDE-2 (37.22%). There are in total 362 controls and 307 patients where 224 (controls)/196 (patients) in ABIDE-1 and 138/111 in ABIDE-2. 13.00% of the participants are female and 87.00% of them are male; 6.73% (female)/56.05% (male) in ABIDE-1 and 6.28%/30.94% in ABIDE-2. The participants are distributed in age ranges 0–20 (33.63%), 20–25 (28.55%), and over 25 (37.82%) years old; 21.97% (0–20)/17.49% (20–25)/23.32% (> 25) in ABIDE-1 and 11.66%/11.06%/14.50% in ABIDE-2.

Available Variables. In the iSTAGING consortium, we have MR imaging (region-of-interest volumes and white matter lesion volume), demographics (gender, age, race and smoking status), clinical (diabetes, hypertension, hyperlipidemia, blood pressure (systolic/diastolic) and body mass index), genetic factor (Apolipoprotein E alleles 2, 3 and 4), and cognitive score (mini-mental state exam) variables. In the PHENOM consortium, we have MR imaging (region-of-interest volumes) and demographics (gender, age, race, education level, marital status, employment status and handedness) variables. In the ABIDE consortium, we have MR imaging (region-of-interest volumes), demographics (gender, age and handedness) variables, and cognitive score (full-scale intelligence quotient, verbal intelligence quotient and performance intelligence quotient) variables.

Methodology for creating features from structural measures

We compute features from T1-weighted MR images using a standard pipeline. Scans are bias-field corrected (8), skull-stripped with a multi-atlas algorithm (9), and then a multi-atlas label fusion segmentation method (10) is used to obtain anatomical region-of-interest (ROI) masks for 119 gray matter ROIs, 20 white matter ROIs and 6 ventricle ROIs of the brain (total 145). We further segment white matter hyperintensities (WMH) using a deep learning-based algorithm (11) on fluid-attenuated (FLAIR) and T1-weighted images. White matter lesion (WML) volumes are obtained by summing up the WMH mask voxels.

Feature pre-processing pipeline. Our data contains structural features such as ROI and WML volumes in addition to demographic, clinical and genetic factors, and cognitive scores. Some of these features (predominantly the last three) are sparsely populated. For continuous-valued features, we first impute missing values with the median of each variable and normalize the feature to have zero-mean and unit-variance. We apply quantile normalization to skewed distributions. For discrete-valued features, we introduce a "unknown" category for missing values. Corresponding to each feature with missing

values, we introduce an additional Boolean feature which indicates whether the value was missing. This way we preserve the evidence of absence (rather than the absence of evidence) (12). We did not use any harmonization tools (13, 14).

Evaluation methodology

We report area under the receiver operating characteristic (AUC) curve on held-out test sets as follows. We split data into 5 equal-sized folds (stratified by labels), use four for training and validation (80%) and the fifth for testing (20%). All hyper-parameter tuning is performed using a further 5-fold cross-validation within the 80% data. This way, the 20% data is a completely independent test set which is used only for reporting the final AUC. We report mean and standard deviation of the AUC over 5 independent test sets (one for each outer fold). This is a computationally expensive, but rigorous, evaluation methodology. The three neurological disorders consist of data from multiple clinical studies; we create the training, validation and test sets for each study independently and then concatenate them.

Hyper-parameter tuning methodology

We compare results from an optimized model, in which hyper-parameter optimization and ensemble learning were performed, with a basic network. For the former, we use a framework called AutoGluon (12) which gives an easy way to train a large number of different types of models (deep networks, k-nearest neighbor classifiers, random forests, CatBoost (15), and LightGBM (16)) and perform hyper-parameter search. For deep network models we create an input layer that concatenates the embedding of continuous-valued and categorical features; the other models can natively handle both these types of features. Using AutoGluon we can also build ensembles of these models via bagging, boosting and stacking. For each neurological disorder, for each of the 5 outer folds, we train the above different types of models using different hyper-parameters in parallel across multiple CPUs and 4 GPUs for 1 hour and build an ensemble that obtains the best classification log-likelihood on the validation data.

The baseline deep network has three fully-connected layers and is also built within the same software framework. This network is trained using data that is normalized to have zero mean and unit standard deviation after dropping missing values. It does not use the pre-processing pipeline described above.

Evaluation on additional fairness metrics

It has been noticed that different fairness metrics are often incompatible. We therefore also assessed fairness using three other metrics. Equal opportunity difference (EO) (17), predictive parity difference (PPD) (18) and generalized entropy index (GEI) (19) are three other well-known group fairness measures. A predictor satisfies "equal opportunity" if the true positive rate (TPR) is the same across sensitive groups. "Predictive parity" is satisfied when the positive predictive value (PPV) is the same for sensitive groups, where PPV is defined as the probability that individuals predicted to belong to the positive class actually belong to the positive class. "Generalized entropy index" is an unified individual and group fairness measure and it also explains how individual fairness and group fairness are related. A value of zero represents perfect equality and higher values denote increasing levels of inequality. We assess fairness of our learned learning models using EO, PPD and GEI with respect to sensitive groups including sex, age, race, and study for all neurological disorders. Our original findings remain unchanged: appropriately designed machine learning models show lower disparities compared to baseline machine learning models under these metrics (except GEI metric for autism spectrum disorder case).

For all three neurological disorders, we find that the ensemble model has small fairness disparities in terms of EO, PPD and GEI on all sensitive groups. For example, for AD, the EO, PPD and GEI of the baseline deep network on held-out data are 0.159 ± 0.029, 0.159 ± 0.028 and 0.073 ± 0.005 across gender respectively, which are larger than those of an ensemble model, 0.043 ± 0.048, 0.029 ± 0.028 and 0.058 ± 0.005 respectively (p-values 3.23×10^{-3}, 1.68×10^{-4} and 2.62×10^{-3}). For SZ, the ensemble shows lower EO, PPD and GEI disparities (0.039 ± 0.042, 0.112 ± 0.060 and 0.102 ± 0.018) compared to the neural net (0.222 ± 0.022, 0.272 ± 0.098 and 0.142 ± 0.024) across age groups with p-values 9.02×10^{-3}, 2.37×10^{-3} and 3.04×10^{-3} respectively. Under EO and PPD metrics, compared to the baseline deep network, the ensemble model has better fairness (p-value < 0.01) for all three disorders except in four cases: race and clinical studies in AD, race in SZ, and clinical studies in ASD. Under GEI metric, the ensemble model has better fairness (p-value < 0.01) across all sensitive attributes for AD and SZ. There's no statistically significant difference in GEI between neural net and ensemble for ASD. We also found that the differences between between ensembles trained only on structural measures and ones trained on multi-source data in terms of these fairness metrics are not statistically significant.

Some practical recommendations for training unbiased machine learning models

We conducted the following experiment for Alzheimer's disease diagnosis using a deep neural net to understand the influence of data pre-processing and hyper-parameter tuning techniques on bias. We performed experiment under the following three settings.

(a) Training without sufficient data pre-processing but with adequate hyper-parameter tuning. This is the case we have shown in the first experiment (baseline deep network). We find that both gender and age sub-groups show biased predictions in this case.

(b) Training with sufficient data pre-processing but without adequate hyper-parameter tuning. We run hyper-parameter tuning for 10× less computer time compared to case (a). We observe that prediction disparity does not appear, and this

holds for all sub-groups. However, this inadequate hyper-parameter tuning leads to relatively poor prediction performance (0.896 in AUC) compared to case (a) (0.924 in AUC).

(c) Training with both adequate data pre-processing and hyper-parameter tuning. This is the setting that we have used for the experiments with the ensemble. As we discussed in the main text, machine learning models do not exhibit biased predictions in this case. This setting also leads to improved AUC, which matches that of case (a).

These ablation experiments suggest that adequate data pre-processing leads to unbiased models which obtain a high AUC. On the other hand, adequate hyper-parameter tuning ensures an accurate model, but it may not provide unbiased predictions. One may therefore ascribe importance to the various factors:data pre-processing, hyper-parameter tuning, ensembling, and multi-source data. Our suggestions for building unbiased and accurate predictive models are as follows.

- Use adequate data pre-processing and hyper-parameter tuning techniques;
- Use ensemble models to obtain robust predictions; there are a number of effective techniques to mitigate to increase in computational complexity of ensembles (20);
- Leverage multi-source data if they are available (21).

References

1. M Habes, et al., The brain chart of aging: Machine-learning analytics reveals links between brain aging, white matter disease, amyloid burden, and cognition in the istaging consortium of 10,216 harmonized mr scans. *Alzheimer's & Dementia* **17**, 89–102 (2021).
2. TD Satterthwaite, et al., Association of enhanced limbic response to threat with decreased cortical facial recognition memory response in schizophrenia. *Am. J. Psychiatry* **167**, 418–426 (2010).
3. T Zhang, N Koutsouleris, E Meisenzahl, C Davatzikos, Heterogeneity of structural brain changes in subtypes of schizophrenia revealed using magnetic resonance imaging pattern analysis. *Schizophr. bulletin* **41**, 74–84 (2015).
4. GB Chand, et al., Two distinct neuroanatomical subtypes of schizophrenia revealed using machine learning. *Brain* **143**, 1027–1038 (2020).
5. A Di Martino, et al., The autism brain imaging data exchange: towards a large-scale evaluation of the intrinsic brain architecture in autism. *Mol. psychiatry* **19**, 659–667 (2014).
6. CR Jack Jr, et al., The alzheimer's disease neuroimaging initiative (adni): Mri methods. *J. Magn. Reson. Imaging* **27**, 685–691 (2008).
7. KA Ellis, et al., Addressing population aging and alzheimer's disease through the australian imaging biomarkers and lifestyle study: Collaboration with the alzheimer's disease neuroimaging initiative. *Alzheimer's & dementia* **6**, 291–296 (2010).
8. NJ Tustison, et al., N4itk: improved n3 bias correction. *IEEE transactions on medical imaging* **29**, 1310–1320 (2010).
9. J Doshi, G Erus, Y Ou, B Gaonkar, C Davatzikos, Multi-atlas skull-stripping. *Acad. radiology* **20**, 1566–1576 (2013).
10. J Doshi, et al., Multi-atlas region segmentation utilizing ensembles of registration algorithms and parameters, and locally optimal atlas selection. *Neuroimage* **127**, 186–195 (2016).
11. J Doshi, G Erus, M Habes, C Davatzikos, Deepmrseg: A convolutional deep neural network for anatomy and abnormality segmentation on mr images. *arXiv preprint arXiv:1907.02110* (2019).
12. N Erickson, et al., Autogluon-tabular: Robust and accurate automl for structured data. *arXiv preprint arXiv:2003.06505* (2020).
13. R Pomponio, et al., Harmonization of large mri datasets for the analysis of brain imaging patterns throughout the lifespan. *NeuroImage* **208**, 116450 (2020).
14. R Wang, P Chaudhari, C Davatzikos, Harmonization with flow-based causal inference. *Int. Conf. on Med. Image Comput. Comput. Interv.* pp. 181–190 (2021).
15. L Prokhorenkova, G Gusev, A Vorobev, AV Dorogush, A Gulin, Catboost: unbiased boosting with categorical features. *Adv. neural information processing systems* **31** (2018).
16. G Ke, et al., LightGBM: A highly efficient gradient boosting decision tree. *Adv. neural information processing systems* **30** (2017).
17. M Hardt, E Price, N Srebro, Equality of opportunity in supervised learning. *Adv. neural information processing systems* **29** (2016).
18. P Garg, J Villasenor, V Foggo, Fairness metrics: A comparative analysis in *2020 IEEE International Conference on Big Data.* (IEEE), pp. 3662–3666 (2020).
19. T Speicher, et al., A unified approach to quantifying algorithmic unfairness: Measuring individual &group unfairness via inequality indices in *Proceedings of the 24th ACM SIGKDD international conference on knowledge discovery & data mining.* pp. 2239–2248 (2018).
20. T Chen, et al., TVM: End-to-end optimization stack for deep learning. *arXiv preprint arXiv:1802.04799* **11**, 20 (2018).
21. JN Acosta, GJ Falcone, P Rajpurkar, EJ Topol, Multimodal biomedical ai. *Nat. Medicine* pp. 1–12 (2022).
